# Effect of continuous compression and 30:2 cardiopulmonary resuscitation on cerebral microcirculation in a porcine model of cardiac arrest

**DOI:** 10.1186/1757-7241-21-55

**Published:** 2013-07-12

**Authors:** Lin Yang, Shuo Wang, Chun-Sheng Li

**Affiliations:** 1 Hyperbaric Oxygen Department, Beijing Chao-Yang Hospital, Affiliated to Capital Medical University, Beijing 100020, China; 2 Department of Emergency Medicine, Beijing Chao-Yang Hospital, Affiliated to Capital Medical University, No. 8, South Road of Worker’s Stadium, Chaoyang District, Beijing 100020, China

**Keywords:** Brain ischemia, Microcirculation, Cardiopulmonary resuscitation, Laser Doppler flowmetry, Haemodynamics

## Abstract

**Background:**

The effect of rescue breathing on neurologic prognosis after cardiopulmonary resuscitation (CPR) is controversial. Therefore, we investigated the cerebral microcirculatory and oxygen metabolism during continuous compression (CC) and 30:2 CPR (VC) in a porcine model of cardiac arrest to determine which is better for neurologic prognosis after CPR.

**Methods:**

After 4 min of ventricular fibrillation, 20 pigs were randomised into two groups (n=10/group) receiving CC-CPR or VC-CPR. Cerebral oxygen metabolism and blood flow were measured continuously using laser Doppler flowmetry. Haemodynamic data were recorded at baseline and 5 min, 30 min, 2 h and 4 h after restoration of spontaneous circulation (ROSC).

**Results:**

Compared with the VC group, the mean cortical cerebral blood flow was significantly higher at 5 min ROSC in the CC group (P<0.05), but the difference disappeared after that time point. Brain percutaneous oxygen partial pressures were higher, and brain percutaneous carbon dioxide partial pressures were lower, in the VC group from 30 min to 4 h after ROSC; significant differences were found between the two groups (P<0.05). However, no significant difference of the cerebral oxygen extraction fraction existed between the two groups.

**Conclusions:**

Inconsistency of systemic circulation and cerebral microcirculation with regard to blood perfusion and oxygen metabolism is common after CPR. No significant differences in cortical blood flow and oxygen metabolism were found between the CC-CPR and VC-CPR groups after ROSC.

## Introduction

The recommendations of the 2010 American Heart Association (AHA) guidelines for cardiopulmonary resuscitation (CPR) are based on adequate chest compressions for a critical amount of blood flow to the brain and the ventilation necessary to achieve adequate gas exchange [1]. Some experimental studies concerning cardiac arrest (CA) have shown that, compared with 30:2 chest compression (VC), continuous compression (CC) presents a similar rate of recovery and better neurological prognosis in addition to no significant deterioration in blood gas parameters in the first 4–8 min of resuscitation [2,3]. In adults with out-of-hospital CA, rescue breathing does not improve survival and neurological prognosis [4-6]. According to the results of our previous study, in the first 12 min of CPR, CC maintains a relatively better coronary perfusion pressure (CPP), PaO_2_ and global ventilation/perfusion than VC [7]. Interruption of chest compressions for rescue breathing resulted in a periodical interruption of vital organ perfusion and lower perfusion pressure at the beginning of each compression cycle [8]. However, other studies have demonstrated that rescue breathing with chest compression achieves a better prognosis than CC alone [9]. Additionally, the tidal volume achieved by CC was found to be practically limited, and the ventilation produced by CC was insufficient to achieve adequate gas exchange [10].

Cerebral blood flow and oxygen delivery are crucial for the capillary exchange beds and are closely related to the recovery of cerebral function after CPR. However, a difference in cerebral microcirculation and oxygen dynamics between CC-CPR and VC-CPR has not been reported. In the present study, we measured blood flow and oxygen delivery in the cerebral cortex to investigate differences in cerebral microcirculation between CC-CPR and VC-CPR in a porcine model of CA.

## Methods

The present study was conducted with the approval of the Animal Care and Use Committee of Chao-Yang Hospital, affiliated with Capital Medical University, in Beijing, China.

### Animal preparation

Twenty male domestic pigs (32±3 kg) were used in this study. Anaesthesia was induced by intramuscular injection of midazolam (0.5 mg/kg), followed by ear vein injection of pentobarbital (8 mg/kg/h) to maintain anaesthesia. A cuffed 6.5-mm endotracheal tube was advanced into the trachea. Pigs were mechanically ventilated by a volume-controlled ventilator (PB-7200; Nellcor Puritan Bennett Incorporated, Pleasanton, CA, USA) with room air using a tidal volume of 15 mL/kg and a respiratory frequency of 12 breaths per minute. The positive end expiratory pressure (PEEP) was set to zero. The end-tidal partial pressure of carbon dioxide (EtPCO_2_) was measured using an in-line infrared capnograph (CO_2_SMO plus monitor; Respironics Incorporated, Pittsburgh, PA, USA). The respiratory frequency was adjusted to maintain EtPCO_2_ between 4.67 and 5.33 kPa before induction of CA and after the return of spontaneous circulation (ROSC). The room temperature was adjusted to 26°C. A Swan-Ganz catheter (7F; Edwards Lifesciences, Irvine, CA, USA) was inserted from the right femoral vein and flow-directed into the pulmonary artery to measure central venous pressure (CVP) and cardiac output (CO) by a Hewlett-Packard monitor (M1165; Hewlett-Packard, Palo Alto, CA, USA). An angiographic catheter was inserted from the femoral artery into the aortic arch for arterial blood pressure (ABP), lactic acid (Lac) and arterial blood gas analyses (GEM Premier 3000 Blood Gas Analyser, Instrumentation Laboratory, Bedford, MA, USA). Electrocardiography (ECG) was conducted with the Hewlett-Packard monitor too. Another catheter (3.8-F) was inserted into the left internal jugular vein and passed in a retrograde direction as far as possible toward the jugular bulb for blood sampling to measure jugular venous oxygen saturation (SjO_2_). This technique was used to allow continuous blood flow in the vein after insertion of the catheter [11]. A 5-F pacing catheter was advanced from the right internal jugular vein into the right ventricle to induce ventricular fibrillation (VF) using a programmed electrical stimulation instrument (GY-600A; Kaifeng Huanan Instrument Limited Company, China). For continuous measurement of cerebral cortical blood flow, both parietal regions of the skull were initially exposed. A laser Doppler flow (LDF) probe (Periflux® Laser-Doppler Flowmeter PF2B; Perimed, Stockholm, Sweden) was placed directly over the surface of the right parietal cortex through a burr hole (1 cm anterior to the coronal suture and 1 cm lateral to the sagittal suture) [12]. The other side was prepared in the same manner for continuous measurement of PaO_2_ and PaCO_2_ of the cerebral cortex.

### Experimental protocol

After surgery, pigs were allowed to acclimatise for 30 min to achieve stability. VF was induced by programmed electrical stimulation [13] and confirmed by ECG with the presence of significant hypotension. Ventilation was stopped, and the ventilator was disconnected from the endotracheal tube. After 4 min of untreated VF, all animals were randomised into two groups with 10 piglets each receiving CC-CPR or VC-CPR [7].

Compressions were performed 100 times/min in both groups. In the VC group, manual ventilation was undertaken twice during a 5-s pause between the two 30 compression cycles using a bag respirator with 300 mL of room air.

After five cycles of CPR, defibrillation (Smart Biphasic) was attempted using 150 J for the first attempt, followed by five cycles of CPR. A 10-s pause was interjected to analyse the rhythm and prepare for the next five cycles of CPR and defibrillation attempts. If an organised cardiac rhythm with a mean aortic pressure ≥60 mmHg persisting for an interval ≥10 min was present, the pigs were regarded to have undergone successful ROSC. Animals without ROSC after four attempted defibrillations were pronounced dead. After successful resuscitation, the pigs were mechanically ventilated using the same parameters as the baseline and then underwent a 4-h intensive care period in which Ringer’s solution (20 mL/kg/h) was administered. The neurological outcomes of the animals were evaluated according to the swine cerebral performance categories (CPCs) at 24 h after ROSC as previously described [14]. The animals were then sacrificed using intravenous potassium chloride and an overdose of pentobarbital to observe the intact lungs.

### Measurements

The mean arterial pressure (MAP), CVP, CO, heart rate (HR), and ECG were recorded. Blood gas analyses of arterial and jugular venous blood were repeatedly performed at baseline, 5 min, 30 min, 2 h and 4 h after ROSC. CO was measured using the thermodilution method by injection of 4°C saline at the same time points. Systemic vascular resistance (SVR) was calculated by the Hewlett-Packard monitor.

The cerebral oxygen extraction fraction (OEF) was calculated using the following formula: OEF = (1 − SjO_2_/SaO_2_) × 100*%*.

Cortical cerebral blood flow (CCBF), brain percutaneous partial pressure of oxygen (PbtO_2_) and brain percutaneous partial pressure of carbon dioxide (PbtCO_2_) were recorded continuously throughout the experiments using the LDF probe. The mean CCBF of the 5 min period was calculated at baseline, 30 min, 2 h and 4 h after CPR. The percentage change of CCBF was calculated using the following formula: Percentage change of CCBF=(PU 2 −PU 1)/PU 1, where PU 1 represented the cortical cerebral blood flow at baseline and PU 2 represented the cortical cerebral blood flow of the observing point. Hereafter, CCBF is used to indicate the percentage change of the cortical cerebral blood flow.

### Statistical analyses

Multivariate ANOVA was used for comparing values between the CC group and VC group, and all variables were considered in a multivariate model and tested for significance. Repeated measures ANOVA was used to compare values at baseline, 5 min, 30 min, 2 h and 4 h after ROSC. The data were reported as the means ± SD. P <0.05 was considered statistically significant. The Statistical Package for Social Sciences (version 13.0; SPSS, Chicago, IL, USA) was used for statistical analyses.

## Results

### Outcomes

Nine pigs of each group received ROSC and survived for 4 h, and there were no significant differences in shocks before ROSC, the duration of CPR before ROSC and 24 h survival between the two groups. The CPCs of the CC and VC groups were 2.99±1.1 and 3.01±1.2, respectively; no significant difference existed between the two groups.

### CCBF and oxygen dynamics

Compared with the VC group, the mean CCBF was significantly higher after ROSC 5 min in the CC group (P<0.05). The mean CCBF subsequently decreased gradually 4 h after ROSC in both groups with no significant difference between the two groups (Table 1).

**Table 1:** Cerebral cortical blood flow and oxygen metabolism of the two groups (mean±SD)

	**Group**	**Baseline**	**ROSC**	**ROSC**	**ROSC**	**ROSC**
			**5 min**	**30 min**	**2 h**	**4 h**
CCBF (%)						
	CC		48±14^a^	−38±11^d^	−40±10^d^	−45±10^d^
	VC		38±11^d^	−43±11^d^	−44±10^d^	−50±12^d^
PbtO_2_ (kPa)						
	CC	9.5±1.3	4.1±0.8^a, d^	8.6±1.2^b, c^	7.0±0.8^a, d^	6.1±0.8^a, d^
	VC	9.8±1.2	3.4±0.8^d^	10.0±1.2	7.8±0.6^d^	6.8±0.8^d^
PbtCO_2_ (kPa)						
	CC	5.3±0.8	5.7±0.8^a^	5.2±0.8^b^	5.3±0.6^b^	5.5±0.5^a^
	VC	5.3±0.5	5.3±0.7	4.1±0.6 ^d^	4.6±0.8^c^	5.1±0.5
PaO_2_ (kPa)						
	CC	11.7±0.9	6.9±0.6^d^	7.3±0.6^d^	9.6±0.9^d^	11.3±0.8
	VC	11.9±0.8	6.5±0.9^d^	7.0±0.7^d^	9.8±0.8^d^	11.7±0.6
PaCO_2_ (kPa)						
	CC	5.8±0.6	5.7±0.4^b^	5.8±0.6^b^	6.0±0.6	6.2±0.7
	VC	5.9±0.7	5.1±0.4^d^	4.5±0.4^d^	5.8±0.5	6.1±0.5
PH						
	CC	7.43±0.03	7.33±0.06^d^	7.35±0.05^d^	7.42±0.04	7.44±0.03
	VC	7.44±0.04	7.35±0.06^d^	7.35±0.04^d^	7.43±0.03	7.45±0.04
Lac (mmol/L)						
	CC	2.03±0.55	5.15±0.58^d^	8.09±1.19^a, d^	6.54±0.70^b, d^	4.09±0.72^a, d^
	VC	1.92±0.58	5.36±0.83^d^	7.26±1.12^d^	5.55±0.86^d^	3.55±0.92^d^
MV (L/min)						
	CC	4.5±0.1	5.6±0.6^d^	4.9±0.4	4.8±0.3	4.8±0.3
	VC	4.4±0.1	5.3±0.5^d^	4.8±0.3	4.8±0.3	4.8±0.2

^a^. p<0.05, ^b^. p<0.01 vs. the VC group; ^c^. p<0.05, ^d^. p<0.01 vs. the baseline.

ROSC: restoration of spontaneous circulation, CC: continuous compression group, VC: 30:2 cardiopulmonary resuscitation group, CCBF: cerebral cortical blood flow (percent change of PU, PU: cortical cerebral blood flow), PbtO_2_: cerebral cortical partial pressure of oxygen, PbtCO_2_: cerebral cortical partial pressure of carbon dioxide, Lac: lactic acid, MV: minute volume.

PbtO_2_ reached a minimum 5 min after ROSC, increased rapidly at 30 min and decreased gradually during the whole period after ROSC in both groups. PbtO_2_ was higher in the VC group than in the CC group, and significant differences were found between the two groups at 5 min, 2 h and 4 h after ROSC (P<0.05). PbtCO_2_ increased from 5 min to 4 h after ROSC. PbtCO_2_ was lower in the VC group, and a significant difference was found between the two groups at each time point after ROSC (P<0.05), whereas no significant difference in MV was found (Table 1). PH and PaO_2_ recovered at 2 h and 4 h after ROSC in both groups (Table 1), and no significant difference in PaO_2_ and pH was found between the two groups. PaCO_2_ was higher at 5 min and 30 min after ROSC in the CC group than in VC group (Table 1). The peak value of Lac appeared at 30 min after ROSC in both groups, and it was higher in the CC group at 30 min, 2 h and 4 h after ROSC than in the VC group (P<0.05).

### Haemodynamic data

MAP was higher at 5 min after ROSC in the CC group than in the VC group. No significant difference was found from 30 min to 4 h after ROSC between the two groups. Cerebral OEF increased maximally at 5 min after ROSC in both groups and then declined to baseline. No significant differences were found between the two groups (Table 2). CVP recovered within 30 min after ROSC in both groups, whereas CO and SVR did not recover until 4 h after ROSC in both groups. No significant difference was found between the two groups (Table 2).

**Table 2:** Haemodynamics of the two groups (mean±SD)

		**Baseline**	**ROSC**	**ROSC**	**ROSC**	**ROSC**
			**5 min**	**30 min**	**2 h**	**4 h**
HR (/min)						
	CC	119±12	152±12^d^	137±15^d^	128±11	120±13
	VC	116±13	149±13 ^d^	127±13	125±13	120±14
MAP (mmHg)						
	CC	101.2±10.0	123.1±10.5^a, d^	98.5±12.5	113.0±10.1^c^	106.0±15.8
	VC	102.3±13.5	111.2±9.4^c^	96.3±14.4	109.7±10.7	106.9±7.7
CO (L/min)						
	CC	4.26±0.44	3.04±0.57^d^	3.18±0.43^d^	3.57±0.43^d^	3.86±0.43
	VC	4.22±0.48	2.88±0.59^d^	3.17±0.56^d^	3.73±0.39^c^	3.88±0.48
CVP (kPa)						
	CC	0.80±0.31	1.20±0.28^d^	0.95±0.16	1.01±0.27	0.81±0.21
	VC	0.91±0.25	1.13±0.24^c^	0.87±0.14	0.88±0.15	0.87±0.15
SVR (mN×s/cm^5^)						
	CC	17.59±2.44	30.26±6.55^d^	22.72±4.60 ^c^	23.25±2.54^d^	21.60±4.76
	VC	17.85±3.15	28.68±5.86^d^	22.64±4.70^c^	21.71±2.19^c^	20.53±3.20
OEF (%)						
	CC	23±8	56±7^d^	38±11^d^	33±12^c^	32±9^c^
	VC	22±7	51±9^d^	44±11^d^	33±10^d^	31±8^c^

^a^. p<0.05, ^b^. p<0.01 vs. the VC group; ^c^. p<0.05, ^d^. p<0.01 vs. the baseline.

ROSC: restoration of spontaneous circulation, CC: continuous compressions group, VC: 30:2 cardiopulmonary resuscitation group, MAP: mean arterial pressure, CO: cardiac output, CVP: central venous pressure, SVR: systemic vascular resistance, OEF: oxygen extraction fraction.

## Discussion

The present study investigated microcirculation after CPR and found inconsistency in the systemic circulation and cerebral microcirculation with regard to blood perfusion. CCBF only increased at 5 min after ROSC, and this increase may have been the result of a transient high MAP and CO in early ROSC. Four hours after ROSC, MAP, CO and CVP had recovered, but the LDF of the parietal cerebral cortex decreased in both groups, demonstrating the inconsistency of the systemic circulation and the cerebral microcirculation with regard to blood perfusion after ROSC. A previous study reported that the delay in continuous multifocal brain hypoperfusion was most obvious 2–12 h after CA, and global cerebral blood flow was reduced to 44–60% of baseline. Local cerebral hypoperfusion includes “no flow”, “trickle flow” and “low flow”, which are scattered and significantly increase ischemia and damage to grey matter [15]. The heterogeneity of the cortical cerebral microcirculation may be a reason why better systemic circulation does not mean better cerebral microcirculation. Vasomotion may be another reason for the inconsistency of systemic circulation and cerebral microcirculation. Vasomotion may exacerbate blood flow and oxygen transport heterogeneity when hypoxia begins, thereby increasing oxygen consumption in the tissue of certain areas [16].

LDF is an appropriate method for continuous measurement of blood flow in the brain; however, blood flow measured using the LDF method shows variability among animals and even within the same animal. The variability is related to the heterogeneity of the brain microcirculation in addition to the temporal and spatial variations of the microcirculation. We used the percentage change in the mean CCBF with respect to baseline values; thus, temporal and spatial variability was reduced, and microcirculation-influenced factors such as cardiac and respiratory cycles and variability in neural activity were controlled for [17,18].

Oxygen delivery mainly depends on local tissue microcirculation parameters, such as tissue PCO_2_, PO_2_, local metabolic byproducts, microcirculatory vasomotion and essential ventilation [16]. PbtO_2_ monitoring is one of the most reliable methods of cerebral oxygen monitoring, which can directly obtain brain tissue oxygen match and metabolic indices. It is a golden standard of therapeutic evaluation [19]. Former research reported PbtO_2_ could predict the mortality and disability rate of craniocerebral injury patients [20], which independently associated with the poor prognosis [21,22]. It could be a bedside auxiliary monitoring to determine brain dead.

In our study, CCBF was higher in the CC group at 5 min after ROSC, which resulted in better cerebral PbtO_2_. Subsequently, CCBF declined during the few hours after ROSC. The reasons for the sharper decrease of PbtO_2_ and higher PbtCO_2_ 5 min after ROSC in the CC group may include insufficient ventilation and transportation of oxygen to local tissues together with accumulation of carbon dioxide, which consequently resulted in anaerobic glycolysis because no significant differences of MV were found between the two groups. Thus, the level of Lac was higher in the CC group. Maintaining only minimum air flow by gasping and passive ventilation through compression-decompression of the chest is not sufficient for metabolism in the brain [2,3]. However, rescue breathing may decrease the dead space after ROSC because of reduced atelectasis by positive pressure ventilation [23]. Because EtPCO_2_ was constant, the smaller gap between PaCO_2_ and EtPCO_2_ in the VC group may have resulted from lower dead space. Another mechanism may be the higher CCBF of the CC group during the initial stages of reperfusion, which can exacerbate neuronal injury through production of free radicals and mitochondrial injury [24,25].

In the CC group, Lac and PbtCO_2_ were maintained at a high level. Serious acidosis may prevent automatic adjustment of the cerebrovascular system, which could explain why CC-CPR provides better systemic circulation but does not result in better blood flow in the cerebral microcirculation and cortical oxygen delivery in the late phase after ROSC. Even with normal blood flow, most of the tissue may be poorly supplied [26]. Acidosis would worsen the central ischemia and hypoxia, producing a vicious circle after CPR. The consistent high level of Lac may result in low transport of oxygen in the CC group. SVR increased during the 4 h after ROSC in both groups, possibly because of peripheral vascular contraction to support the oxygen supply of the brain after CPR. CC-CPR may not alter microvascular smooth muscle tone and may thus not redistribute blood within the microvascular bed, in contrast to VC-CPR.

We also monitored cerebral OEF, which is an indicator of global cerebral oxygen uptake. There were no significant differences in OEF between the two groups during the 4 h after ROSC; there was an extremely high rate of OEF in both groups. One possible explanation may be that cerebral vessels dilate because of increased PaCO_2_ after CPR, thus leading to increased availability of oxygen in brain tissue [27].

### Study limitations

The holes created in the parietal bone during craniotomy could influence the intracranial pressure; however, we did not record intracranial pressure in the present study. Intracranial pressure influences cerebral perfusion and the adjustment of the microcirculation. Detecting cerebral hypoxic-ischaemic events after CPR is difficult, particularly in parenchymal tissues such as the brain. The metabolism of the microcirculation and tolerance to anoxia are inconsistent in different brain regions. In the present study, the parietal cortex was chosen for detection of CCBF. Because both groups experienced the same protocol, these biases do not influence our conclusions.

## Conclusions

Inconsistency of the systemic circulation and cerebral microcirculation with regard to blood perfusion and oxygen metabolism is common after CPR. Both CC-CPR and VC-CPR have the same prognosis. VC-CPR presents better PbtO_2_ and PbtCO_2_ at 5 min after ROSC, although no significant differences in CCBF and oxygen metabolism after CPR were found between CC-CPR and VC-CPR.

### Key messages

Cerebral blood flow and oxygen delivery are crucial for the capillary exchange beds and are closely related to the recovery of cerebral function after CPR.

Inconsistency between the macrocirculation and cerebral microcirculation is observed after CPR following cardiac arrest.

Both CC-CPR and VC-CPR present the same prognosis.

VC-CPR presents better PbtO_2_ and PbtCO_2_ after ROSC.

There are no significant differences in cortical blood flow and oxygen metabolism after CPR between CC-CPR and VC-CPR.

## Abbreviations

CPR: Cardiopulmonary resuscitation; CA: Cardiac arrest; VC: 30:2 chest compression; CC: Continuous compression; CPP: Coronary perfusion pressure; EtPCO2: End-tidal partial pressure of carbon dioxide; ROSC: Return of spontaneous circulation; CVP: Central venous pressure; CO: Cardiac output; ABP: Arterial blood pressure; Lac: Lactic acid; ECG: Electrocardiography; SjO2: Jugular venous oxygen saturation; VF: Ventricular fibrillation; LDF: Laser Doppler flow; CPC: Cerebral performance category; MAP: Mean arterial pressure; HR: Heart rate; SVR: Systemic vascular resistance; OEF: Cerebral oxygen extraction fraction; CCBF: Cortical cerebral blood flow; PbtO2: Percutaneous partial pressure of oxygen; PbtCO2: Percutaneous partial pressure of carbon dioxide.

## Competing interests

The authors declare that they have no competing interests. The manuscript, including related data, figures and tables, has not been published previously and is not under consideration elsewhere.

## Authors’ contributions

LY: conception and design of the research, drafting of the manuscript and revising it critically for important intellectual content; SW: acquisition, analysis and interpretation of data; C-SL: giving final approval of the version to be published. All authors read and approved the final manuscript.
